# Incidence and outcome of osteonecrosis in children and adolescents after intensive therapy for acute lymphoblastic leukemia (ALL)

**DOI:** 10.1002/cam4.645

**Published:** 2016-01-20

**Authors:** Bhavna Padhye, Luciano Dalla‐Pozza, David Little, Craig Munns

**Affiliations:** ^1^ Department of OncologyThe Children's Hospital at WestmeadSydneyAustralia; ^2^ Department OrthopaedicsThe Children's Hospital at WestmeadSydneyAustralia; ^3^ Department of EndocrinologyThe Children's Hospital at WestmeadSydneyAustralia

**Keywords:** ALL, osteonecrosis, outcome

## Abstract

Osteonecrosis (ON), a significant complication following treatment of acute lymphoblastic leukemia (ALL), has a profound impact on quality of life of ALL survivors. We studied incidence and outcome of ON in patients treated on or according to Australian and New Zealand Children's Haematology/ Oncology Group (ANZCHOG) study 8 at The Children's Hospital at Westmead. The study involved retrospective chart review of the patients. ON was defined by development of symptoms and confirmed by magnetic resonance imaging. From 2002–2011, 251 patients (143M, 108F, 59 Standard Risk (SR), 159 Medium Risk (MR) 5 High Risk (HR), and 28 Very high risk (VHR)) were treated according to study 8. Eighteen (7M, 11F, 2 SR, 12 MR, 4 VHR) patients developed ON (7.2%). Median age at diagnosis was 13.05 years(4.3–16.7). Incidence of ON in patients > 10 years at diagnosis was 29%. Six out of 18 patients developed ON after allogeneic stem cell transplantation. Median time from diagnosis to the development of ON following chemotherapy for ALL was 1.15 years (range 0.25–2.12). Most patients were treated with intravenous Zoledronic acid. At last follow‐up, three patients had undergone arthroplasty, two patients were symptom free, and the remaining 13 patients reported persistent pain with activity. A majority of patients with ON of the hips had radiological progression. Overall, 7% of patients with ALL developed ON. Age >10 years was the most important risk factor. At last follow‐up, 70% of patients had persistent symptoms. Although Zoledronic acid improved pain, most patients with ON of the hips had radiological progression.

## Introduction

As survival rates for childhood acute lymphoblastic leukemia (ALL) improve, awareness, treatment, and prevention of adverse effects becomes increasingly important 1, 2 Osteonecrosis (ON) is a well‐recognized complication of chemotherapy in children and adolescents with ALL and it significantly impacts on the long‐term quality of life. The Childhood Cancer Survivor Study has compared the rate of self‐reported ON in cancer survivors with the rate observed in a sibling comparison group. The rate ratio was 6.5 in patients with ALL treated with chemotherapy, 11.2 in chemotherapy‐treated patients with acute myeloid leukemia (AML), and 59.2 for patients undergoing allogeneic hematopoietic stem cell transplantation (HSCT) recipients for leukemia 3. Glucocorticoid use (prednisolone and dexamethasone) has been implicated as a major etiological factor in the development of ON in this patient group. Other risk factors include age >10 years, female sex, Caucasian race, and higher body mass index 4, 5, 6, 7, 8. Some ALL treatment regimens have a much higher frequency of ON than others, suggesting that some nonglucocorticoid drugs (e.g., Asparaginase and methotrexate) may modify the risk of osteonecrosis 9.

In addition genetic factors have a role in predisposing individuals to ON. Multiple candidate genes studies and GWAS have indicated several polymorphisms in genes putatively related to the development of ON such as *SERPINE1, VDR, CYP3A4, ACP1*,* and SH3YL1* 4, 10, 11, 12, 13.

We studied incidence and outcome of ON in patients with ALL treated in a single institution on the Australian and New Zealand Children's Haematology Oncology group (ANZCHOG) ALL Study 8 (ANZCHOG Study 8) protocol at The Children's Hospital at Westmead, Sydney, Australia.

## Patients and Methods

### Study population and treatment

From 2002–2011, 251 children and adolescents with newly diagnosed ALL or lymphoblastic lymphoma were enrolled and treated on (Patients with ALL) or according to (patients with lymphoblastic lymphoma) the ANZCHOG study 8. (The ANZCHOG ALL8 trial is registered on the Australian and New Zealand clinical Trials registry as ACTRN12607000302459 http://www.anzctr.org.au/trial_view.)

Patients on this trial were stratified into risk groups using minimal residual disease (MRD) levels at day 33 and day 79 together with other biological and clinical risk factors 14. Patients were divided into standard, medium, high, and very high‐risk groups. The very high‐risk (VHR) group was defined as patients with one or more of following factors: high levels of MRD at day 79, prednisolone poor response (PPR), and T‐cell ALL or white cell count (WCC) > 100 × 10^9^/L, BCR‐ABL1 positive ALL or positive for the MLL t (4, 11) translocation, not in morphological remission at day 33, M2 bone marrow at day 15 .The high‐risk (HR) group had all of the following features: MRD <10^−3^ at day 79, PPR (absence of T‐cell ALL, t (9, 22) and t (4, 11), morphological remission at day 33. The standard‐risk group (SR) had no high‐risk features and were MRD negative at both day 33 and day 79 using two MRD markers with a minimum sensitivity of 10^−4^. The medium‐risk (MR) group were patients not qualifying for either standard or high risk. The stratification was the same as AEIOPBFM ALL‐2000 and there were no randomizations in the protocol 15. SR and MR patients were treated uniformly according to the common control arm in BFM ALL‐2000. High‐risk patients were assigned to treatment with novel high‐risk chemotherapy blocks 16.

Of 251 patients, 48 patients underwent stem cell transplantation. Following completion of treatment, all patients were followed up in oncology clinic and then referred to long‐term follow‐up unit to monitor for late side effects of treatment.

### Osteonecrosis

Retrospective chart analysis was undertaken to identify patients with ON. For patients receiving chemotherapy only, ON was defined by development of pain in bones/joints while on treatment or within 1 year of completion of treatment and confirmed by magnetic resonance imaging (MRI). X‐rays of the affected joints were performed at baseline and then 3–6 monthly as clinically indicated. Hip X‐rays were scored using the Association Research Circulation Osseous (ARCO) international classification of ON 17. Similar scoring system was used for knee and ankle ON. Stage I—abnormal MRI or bone scan but normal X‐ray, stage II—abnormal X‐ray showing sclerosis but no collapse, stage III—early collapse or deformity and stage IV—joint destruction.

All patients were seen by orthopedic surgeons and treatment included analgesia, periods of nonweight bearing, bisphosphonates, and surgical procedures including joint replacement in some patients.

For outcome assessment, clinical symptoms and radiological findings (X‐ray and or MRI) at the last follow‐up were recorded. Based on last follow‐up, patients were classified symptomatically as being pain free, having mild to moderate pain without restricting activities and having pain restricting activities and hence undergone joint replacement surgery.

## Results

### Patients

From 2002 to 2011, 251 (143 males and 108 females) patients were treated for ALL/lymphoblastic lymphoma as per ANZCHOG study 8 at The Children's Hospital at Westmead. Fifty‐five (22%) patients were over 10 years at diagnosis. The numbers in each risk stratification grouping was as follows: SR 59, MR 159, HR 5, and VHR 29. Of 251 patients, 48 patients underwent stem cell transplantation either in first remission (*n* = 21) or following relapse (*n* = 24) or for therapy‐related acute myeloid leukemia/myelodysplastic syndrome (AML/MDS) (*n* = 3).

### Incidence of osteonecrosis

Eighteen patients developed ON (Overall incidence 7%).Incidence of ON in females was 11.2% and that in males was 5.2% (*P *= 0.12). Incidence of ON in patients older than 10 years at diagnosis is 29%. Median age at diagnosis of patients who developed ON was 13.05 years (4.3–16.7). Age more than 10 years is a significant risk factor for development of ON (*P* < 0.00001, Chi ‐squared test).

According to treatment group, 3.4% patients in SR, 7.5% in MR, and 13.8% in VHR group developed ON. Three out of four VHR patients with ON underwent stem cell transplantation and required post‐transplant GVHD therapy. No patient in HR group developed ON. Six patients developed ON following allogeneic stem cell transplant (12.5%).

Median time from diagnosis to the development of ON following chemotherapy for ALL was 1.15 years (range 0.25–2.12). Most patients who developed ON following stem cell transplantation (SCT) developed it within 1 year of SCT. There was no difference in the incidence of ON between patients with T‐cell ALL and Pre‐B ALL.

### Chemotherapy and exposure to steroids

All patients underwent uniform induction therapy with prednisolone, vincristine, asparaginase, daunorubicin, and intrathecal methotrexate. SR, MR, and HR patients not undergoing SCT had reinduction course with dexamethasone. Cumulative steroid exposure for this group of patients was 3413 mg/m^2^. (1 mg dexamethasone = 6.67 mg prednisolone)^30^. All SR and MR patients received 4 courses of high‐dose methotrexate during protocol M. HR and VHR patients received 3–6 blocks of intensive therapy consisting of varying drugs. VHR patients underwent allogeneic BMT if there was a suitable donor available. Maintenance therapy consisted of daily 6‐mercaptopurine and weekly oral methotrexate to complete 2 years of therapy from diagnosis. As most of the patients developed symptomatic ON after completion of protocol 2 (reinduction), steroid dose was not modified.

### Stem cell transplant

Six patients developed ON following allogeneic SCT. Two patients underwent transplant in first remission for VHR disease and four patients underwent transplant following relapse of ALL. All four patients received steroid containing reinduction treatment prior to transplant. All patients received total body radiation as part of conditioning for transplant. Five out of six patients developed acute graft versus host disease (aGvHD) post‐transplant requiring steroid therapy.

### Features of osteonecrosis

All patients presented with pain in the joints while on chemotherapy or soon after finishing intensive phase of chemotherapy. Pain in the hips or knees was the most common complaint (88%). Diagnosis of osteonecrosis was confirmed after evaluating bone scans, MRI, and X‐rays of the affected joint. Most patients had involvement of multiple joints and lower limb joint were involved in all patients except one who had isolated shoulder involvement. At diagnosis of ON, X‐rays of affected joints in 15/18 patients (83%) showed minimal (ARCO II) or no change (ARCO I). Remaining three patients showed evidence of joint deformity (ARCO III) on initial X‐ray. No patient presented with joint collapse at initial diagnosis (Table 1).

**Table 1:** Features of osteonecrosis and outcome

Patient Gender/Age (years)	Risk group and treatment	Joint involved – most symptomatic joint underlined	Radiological grading of most symptomatic joint at diagnosis (ARCO)	Radiological grading of most symptomatic joint on latest radiograph (ARCO)	Final clinical outcome at last follow‐up^a^
1. M/12.5	MR	Knees	II	II	1
2.F/14.5	MR	Hips	I	III	3
3.F/13.2	MR	Knees	II	III	2
4.M/13.9	MR	Knees, Hips	II	II	2
5.M/14	MR	Knees, L elbow	III	IV	2
6.F/16.7	SR	Hips, knees, L ankle	I	I	2
7.F/10.6	VHR	Shoulders, Hips, Knees, R ankle	II	II	1
8.F/12.9	MR	Knee	III	III	2
9.F/11.6	MR	Hips, Knees	III	III	2
10.F/8.4	SR	Knees, Ankles	II	II	2
11.F/11.3	VHR SCT in first remission	Hips, knees	I	IV	2
12.M/15.2	VHR SCT in first remission	Knees, ankles, shoulders	I	II	2
13.M/4.3	MR SCT following relapse	Ankle	II	II	2
14.F/16.4	MR SCT following Relapse	Shoulder	II	II	2
15.F/10.6	MR SCT following relapse	Shoulders, Knees, Hips, L Ankle	II	IV	3
16.F/13.3	MR SCT following relapse	Hips	II	IV	2 (Dead)
17.M/14.6	MR SCT following relapse	Knees, Ankles	I	II	2 (Dead)
18.M/12.1	VHR SCT therapy‐related MDS/AML	Hips, Knees, Ankles	I	IV	3(Dead)

AML, acute myeloid leukemia; ARCO, Association Research Circulation Osseous; VHR, very high risk; MDS, myelodysplastic syndrome; MR, medium risk; SCT, stem cell transplantation; SR; standard risk

a 1 = pain free, 2 = having mild to moderate pain not restricting activities, and 3 = having pain restricting activities and hence undergone joint replacement surgery.

Steroid therapy was modified in only one patient after diagnosis of ON. This patient, who developed ON early during therapy, received intermittent dexamethasone (7 days on and 7 days off) rather than the protocol specified continuous 21 day dexamethasone during reinduction. All other patients completed planned treatment with steroids.

### Treatment of osteonecrosis

All patients were reviewed and managed by a single orthopedic surgeon. Therapy consisted of nonweight bearing for a variable period of time. Pain was managed with oral analgesia in consultation with Pain and Palliative care services at the hospital. Following review by a pediatric endocrinologist, all except two patients were treated with intravenous Zoledronic acid (ZA). One of the two patients had abnormal renal function and the other had isolated involvement of shoulder joint. Prior to treatment with ZA, all children were evaluated by a pediatric dentist and any necessary dental work was performed before commencing ZA treatment. ZA was administered 0.025 mg/kg/dose at 12 weekly interval diluted in 50 mL of 0.9% Nail infused over 30 min. Median number of ZA doses per patient was 6 (range 2–8). Most common side effect of ZA infusion was acute flu‐like symptoms after first dose .No patient developed significant hypocalcemia (<2 mmol/L). Bone mineral density and bone turnover were monitored at regular intervals.

### **Outcome of osteonecrosis**

(Table 1) At diagnosis, 6 of 18 patients had predominant involvement of hip joints. All these patients progressed to ARCO stage III or IV at last follow‐up despite treatment and three have undergone joint replacement surgery for increasing pain. One other patient died prior to planned surgical intervention. Two other patients report mild moderate pain and radiologically show deformed joint potentially requiring future hip replacement.

Six patients had predominant involvement of knee joints at diagnosis. At last follow‐up, one patient is pain free. Radiologically, three patients have minimal or no joint destruction and three patients have evidence of joint deformity. No patient has undergone joint replacement surgery. One patient has undergone arthroscopy for removal of foreign bodies from the joint and other has undergone patella chondroplasty.

Four patients had predominant involvement of ankle joints at diagnosis. At last follow‐up, all patients report mild pain in the joint and radiologically show sclerosis but no joint collapse (ARCO II).

Two patients had predominant involvement of shoulder, including one with isolated shoulder involvement who was not treated with ZA. At last follow‐up, both patients have mild to moderate pain and evidence of sclerosis on the radiographs. (ARCO II).

## Discussion

The incidence of symptomatic osteonecrosis for children with ALL or lymphoblastic lymphoma treated as per on ANZCHOG study 8 is 7% for the whole group and 29% for children more than 10 years at diagnosis. Study 8 included prednisolone 60 mg/m2/day for 28 days (tapered over 9 days) during induction and dexamethasone 10 mg/m^2^/day for 21 continuous days (tapered over 9 days) during reinduction. There were no steroid pulses during maintenance.

Study 8 treatment protocol was similar to BFM 95 and BFM 2000, but the incidence of ON was significantly greater in Study 8. A possible explanation for this is better reporting and completeness of the data as this is single‐institution study. Burger et al. reported incidence of ON based on spontaneous reporting to the study center and via questionnaire, from all 64 participating centers for patients treated according to BFM 95 protocol (*n* = 1951). The overall 5‐year cumulative incidence for ON is 1.8%. The incidence increased significantly with age, however, with patients >10 years having an incidence of 8.9% and patients >15 years an incidence of 16.7% 16. The successor trial BFM 2000 study collected data on ON prospectively, incidence of ON in patients >10 years of age was 18.4% for females and 7.6% for males. The authors concluded that as the control treatment arms of the two trials were quite similar and the randomized treatment modifications in ALL‐BFM 2000 had no influence on the ON rate, the higher incidence may be attributed to the prospective data collection in this study and possibly to a higher attention to these complications by the attending doctors 18.

The reported incidence of osteonecrosis in the literature is variable. 7, 8, 18, 19, 20, 21, 22, 23, 24, 25, 26, 27, 28 It is difficult to compare the incidence due to definitions of ON, differences in the treatment protocol, and glucocorticoid use (prednisolone vs. dexamethasone, continuous vs. discontinuous steroids, intensity of other drugs, e.g., asparaginase and cumulative dose of steroids including pulses during maintenance) and retrospective nature of most studies. In a prospective study at St. Jude's Children's Hospital and Research Centre (SJCHRC), the cumulative incidence of any versus symptomatic ON was 71.8% versus 17.6%, respectively. Of those patients older than 10 years of age, 44.6% developed symptomatic osteonecrosis compared with 10% in younger patients^[12]^.

Glucocorticoids (prednisolone and dexamethasone) appear to be the main etiological factors for development of ON. It is reported that dexamethasone, especially when administered continuously, is more toxic to bones as compared to prednisolone, although there are conflicting reports in the literature. 29, 30 Other drugs like asparaginase can influence steroid exposure and hence indirectly affect incidence of ON 31. Genetic polymorphisms in many genes including *SERPINE1, VDR, CYP3A4, PAI 1, and ACP 1* are reported to be associated with ON. A recent meta‐analysis of genetic risk factors for glucocorticoid‐induced osteonecrosis concluded that PAI‐1 4G/5G and ABCB1 C3435T polymorphisms are risk factors for ON 32. Other risk factors include age >10 years, female sex, Caucasian race, higher body mass index, low albumin, and high cholesterol. In our study, age was most significant risk factor for development of ON.

The natural history of ON in the pediatric population and the factors that predict long‐term outcome are not well defined. Older age at diagnosis, pain at presentation, area of involvement (>30%), and lesions close to articular surface has been associated with joint collapse and need for arthroplasty. A recent study at SJCHRC described utility of early screening MRI for extensive hip osteonecrosis in pediatric ALL patients more than 10 years of age at diagnosis. On the basis of prospectively acquired protocol‐driven MRI of the hips obtained after standardized exposure to glucocorticoids, they found osteonecrosis in 17.1% of patients within 1 year of starting glucocorticoids and in 21.7% of patients by the end of ALL therapy, such that 79% of patients who would ultimately develop osteonecrosis did so within 1 year of starting glucocorticoids 33. In our study, the median time for diagnosis of symptomatic ON for chemotherapy patients was 1.15 years. Patients who developed ON following allogeneic SCT developed it within 1 year of transplant and majority of these patients (5/6) had developed acute graft versus host disease requiring prednisolone for treatment. We do not know if these patients had developed asymptomatic changes of ON prior to SCT. It is currently unknown if medical intervention at an earlier stage would improve joint outcome. Also, there is no consensus about how best to manage glucocorticoid therapy in patients who develop ON. The underlying diagnosis, phase of treatment, severity of the disease, and severity of joint involvement needs to be considered. For patients with joints that have already sustained grade III or IV damage, it is unlikely that discontinuation of steroids will change natural history of ON. In our study group, as the majority of patients were diagnosed after reinduction phase, glucocorticoid therapy was unaltered in all but one patient (who received discontinuous dexamethasone).

The only strategy proven in a randomized controlled trial to reduce the development of symptomatic ON is avoidance of prolonged, continuous exposure to dexamethasone. In the Children's Cancer Group 1961 trial in high‐risk ALL, 823 patients of age 10–21 years at diagnosis who had a rapid early response to induction chemotherapy were randomly assigned to receive dexamethasone 10 mg/m^2^ per day on days 0 through 20 of delayed intensification versus 10 mg/m^2^ per day on days 0 through 6 and 14 through 20. The cumulative incidence of symptomatic osteonecrosis at any site was 17% in patients treated with 21 days of continuous dexamethasone, but only 8.7% in those treated with the interrupted schedule (hazard ratio, 2.1; *P* < 0 .001) 34.

Current treatment for ON includes analgesia, limited weight bearing and various surgical procedures including joint replacement. A number of medical therapies have also been undertaken, with variable and inconsistent results including hyperbaric oxygen, nifedipine, prostaglandin infusions, low‐molecular weight heparin, and statins. Few pediatric studies have reported use of bisphosphonates for chemotherapy‐related ON. In an observational study of 17 children with ON as a complication of chemotherapy for leukemia, improvements in the pain scores, analgesic requirement, and function were found in the nine patients who received bisphosphonate therapy while seven of eight patients who did not receive therapy showed clinical deterioration 35.

Another small study of childhood leukemic patients found a reduction in pain and increased mobility in four of the six patients treated with Pamidronate for 2 years; however, three of the six patients had a progression and required hip replacement 36. In our study, Zoledronic acid treatment improved pain in most patients. We also noted that patients who had predominant involvement of knees, ankles, or shoulder joint tended to stabilize after initiating treatment with ZA. All patients with predominant hip joint involvement showed radiological progression. It is currently unknown if there are differences in the natural history of hip and other joint ON. In addition, small lesions could improve spontaneously which makes evaluation of effectiveness of any nonsurgical intervention difficult. Patients who have grade III or IV joints at the end of treatment are more likely to develop early osteoarthritis and require joint replacement in early adult life. The relative risk of joint replacement in long‐term survivors of child hood cancer, as compared with siblings is 54 (95% CI 7.6–386.3) 37. A major concern with replacing joints in young patients is the durability of current prostheses and the probable need for replacement after 10–15 years.

As the outcome of ON with current medical therapies, especially involving hip joints is poor, future studies are needed to identify patients at high risk of this complications and possible intervention to change the natural history. All studies have identified age more than 10 years as a risk factor, if genetic markers described in the literature are confirmed a more targeted approach for screening and early intervention could be developed. One potential but likely reason for relative poor effectiveness of ZA in preventing joint collapse following ON is poor distribution to necrotic bone. Animal studies performed at our institution have shown that prophylactic administration of ZA improves femoral head architecture in rat models of ON 38, 39. While this strategy would not prevent the development of ON, this is an attractive strategy to prevent joint collapse and destruction of an effected in children and adolescents at high risk of chemotherapy‐related ON, without compromising the intensity of chemotherapy.

## Conflict of Interest

No conflict of interest.
